# Phosphatidylinositide 3-Kinase Contributes to the Anti-Inflammatory Effect of* Abutilon crispum* L. Medik Methanol Extract

**DOI:** 10.1155/2018/1935902

**Published:** 2018-11-26

**Authors:** Stephanie Triseptya Hunto, Kon Kuk Shin, Han Gyung Kim, Sang Hee Park, Junsang Oh, Gi-Ho Sung, Mohammad Amjad Hossain, Ho Sik Rho, Jongsung Lee, Jong-Hoon Kim, Jae Youl Cho

**Affiliations:** ^1^Department of Integrative Biotechnology, Sungkyunkwan University, Suwon 16419, Republic of Korea; ^2^Institute for Healthcare and Life Science, International St. Mary's Hospital and College of Medicine, Catholic Kwandong University, Incheon 22711, Republic of Korea; ^3^Department of Veterinary Physiology, College of Medicine, Chonbuk National University, Iksan 54596, Republic of Korea; ^4^Department of Chemical Engineering, College of Engineering, Suwon University, Suwon 18323, Republic of Korea

## Abstract

*Abutilon crispum* L. Medik, better known as bladdermallow, is used as a traditional remedy in India, for its anti-inflammatory effect due to its high content of flavonoids. However, research about its anti-inflammatory effect at the molecular level has not been performed. In this study, we aimed to investigate the mechanism of* Abutilon crispum* methanol extract (Ac-ME) in inhibiting the inflammatory response by conducting several experiments including cellular and molecular assays. Ac-ME inhibited the production of nitric oxide (NO) in RAW264.7 cells during treatment of LPS and Pam3CSK4 without exhibiting cytotoxicity. Ac-ME also suppressed the mRNA expression of inducible nitric oxide (iNOS) and proinflammatory cytokines such as interleukin (IL)-1*β* and IL-6. Moreover, Ac-ME was shown to inhibit the NF-*κ*B pathway, according to the luciferase reporter gene assay performed with a NF-*κ*B-Luc construct containing NF-*κ*B-binding promoter regions under MyD88 and TRIF overexpression conditions, and immunoblotting analysis by determining the phospho-form levels of I*κ*B*α*, IKK*α*/*β*, and p85, a regulatory domain of phosphatidylinositide 3-kinase (PI3K). Finally, we observed that the level of phospho-p85 induced by the overexpression of spleen tyrosine kinase (Syk) and Src was decreased by Ac-ME at 200 *μ*g/ml. Therefore, these results suggest that Ac-ME has an anti-inflammatory effect by targeting PI3K in the NF-*κ*B signaling pathway.

## 1. Introduction

When the body is invaded by foreign organisms and toxins, it will provide protection in the form of an immune system response. The innate immune system is the first to react rapidly and perform an inflammatory response [1, 2], characterized by redness, swelling, and pain. The inflammatory cascade begins when the signature components of pathogens, known as pathogen-associated molecular patterns (PAMPs), interact with pattern recognition receptors (PRRs). Toll-like receptors (TLRs), one of the subclasses of PRRs, can recognize specific PAMPs from viruses, bacteria, fungi, and protozoa. For instance, TLR 2 recognizes peptidoglycans from gram positive bacteria, while TLR 4 recognizes LPS from gram-negative bacteria. The interactions of TLRs and PAMPs lead to recruitment of adaptor proteins such as MyD88, TRIF, TRAM, and TIRAP, which can mediate another cascade effect [3, 4]. This cascade effect involves the signaling pathway leading to activation of transcription factors. The signal mainly consists of NF-*κ*B, AP-1, and IRF-3 pathways. The NF*κ*B pathway is activated when the cascade passes through protein tyrosine kinases (Syk and Src), serine/threonine kinases (AKT), phosphatidylinositide 3-kinases (PI3K), and I*κ*B kinases (IKK). It is called the NF-*κ*B pathway due to involvement of the NF-*κ*B protein (the transcription factor), which is translocated to the nucleus. Translocation of this transcriptional factor will eventually lead to production of inflammatory genes (iNOS and other cytokines) and the release of proinflammatory mediators (NO, interleukins (IL-1), TNF-*α*, and PGE_2_) [5].

Even though inflammation is important for the host defense against infections, it also may contribute to many chronic diseases. Tissue injury, oxidative stress, angiogenesis, and fibrosis are the results of a series of inflammatory responses that may lead to other deadly complications such as cancer [6, 7]. Therefore, development of new treatment to prevent and treat these diseases is essential. Herbal remedies are becoming popular for treating inflammatory diseases, but some of them remain untested.* Abutilon crispum* L. Medik, better known as* Herissantia crispa* L. Brizicky, is a trailing perennial shrub of the family Malvaceae and is known in America as bladdermallow. Traditionally, it is used in India for treatment of asthma, cough, diabetes, ulcers, and jaundice [8]. This plant is rich in flavonoids but has not been well studied with regard to pharmacology [9]. One of the studies found that* Abutilon crispum* L. Medik can be used as an anti-inflammatory agent through* in vivo* experiments, but the exact mechanism remains unknown [10]. Therefore, we aimed to identify the molecular target of the methanolic extract of* Abutilon crispum* L. Medik with a special focus on the NF-*κ*B signaling pathway.

## 2. Materials and Methods

### 2.1. Materials

The methanol extract (code no: PBID 10699) of* Abutilon crispum* L. Medik (Ac-ME) was purchased from International Biological Material Research Centre (http://www.ibmrc.re.kr, Daejeon, Korea). Pam3CSK4, lipopolysaccharide (LPS, E. coli 0111:B4), 3-(4,5-dimethylthiazol-2-yl)-2,5-diphenyltetrazolium bromide (MTT), polyethyleneimine (PEI), sodium dodecyl sulfate (SDS), and dimethyl sulfoxide (DMSO) were purchased from Sigma Chemical Co. (St. Louis, MO, USA). PP2 and Piceatannol (Picea) were purchased from Calbiochem (La Jolla, CA, USA). Fetal bovine serum (FBS), penicillin/streptomycin, TRIzol, DMEM, and RPMI 1640 were obtained from GIBCO (Grand Island, NY, USA). RAW264.7 (ATCC No.: CRL-2278) and HEK293 (ATCC No.: CRL-1573) cells were products from American Type Culture Collection (Rockville, MD, USA). Primers used for semiquantitative reverse transcriptase polymerase chain reactions (RT-PCR) were obtained from Macrogen Inc. (Seoul, Korea). Luciferase constructs with NF-*κ*B binding sites and epitope-tagged signaling expression constructs (FLAG-MyD88, CFP-TRIF, HA-Src, and Myc-Syk) were used as previously reported [11]. All other chemicals were Sigma grade. Phospho-specific and total antibodies recognizing the NF-*κ*B family were obtained from Cell Signaling Technology (Beverly, MA, USA).

### 2.2. Cell Culture and Drug Preparation

RAW264.7 cells were maintained in RPMI 1640 supplemented with 10% heat-inactivated FBS and 1% penicillin/streptomycin, while HEK293 cells were cultured in DMEM media complemented with 5% heat-inactivated FBS and 1% penicillin/streptomycin at 37°C in 5% CO_2_. For each experiment, RAW264.7 and HEK293 cells were detached with cell scrappers and trypsin, respectively. The cells were counted with Trypan blue dye exclusion tests to achieve the desirable experimental cell density. Stock solutions of Ac-ME, Pam3CSK4, PP2, and piceatannol were prepared in DMSO for the* in vitro* experiments.

### 2.3. HPLC Analysis of Ac-ME

The constituents of* Abutilon crispum* L. Medik in methanol extract were analyzed using high-performance liquid chromatography (HPLC), as reported previously [12]. The standard compounds used in this analysis were quercetin, luteolin, and kaempferol. The conditions used in the HPLC analysis are described in Table 1.

### 2.4. Measurement of NO Production

RAW264.7 macrophage cells were maintained for 18 hours, pretreated with Ac-ME (0-200 *μ*g/ml) for 30 minutes, and further incubated with LPS (1 *μ*g/ml) for 24 hours. The inhibitory effects of Ac-ME on production of NO were determined by interpreting the NO level using Griess reagents and measured using a Synergy HT Multi-Mode Microplate Reader (BioTek Instruments GmbH, Bad Friedrichshall, Germany) as previously reported [13].

### 2.5. Cell Viability Test

RAW264.7 or HEK293 cells were plated with a density of 1 × 10^6^ cells/ml, preincubated for 18 hours, and then treated with Ac-ME (0-200 *μ*g/ml) for an additional 24 hours. The cytotoxic effect of Ac-ME was tested by a conventional MTT assay, as previously reported [14, 15]. MTT solution (10 mg/ml in phosphate-buffered saline, pH 7.4, 5 *μ*g/ml as a final concentration) was added, and the cells were continuously cultured for three hours. The incubation was terminated by the addition of 15% SDS into each well, solubilizing the formazan that was then measured using a Synergy HT Multi-Mode Microplate Reader with 570 nm absorbance (OD_570-630_).

### 2.6. mRNA Precipitation and Analysis Using Semiquantitative Reverse Transcriptase Polymerase Chain Reactions

RAW264.7 cells were plated with a density of 1 × 10^6^ cells/ml, preincubated for 18 h, and then treated with Ac-ME (0-200 *μ*g/ml) in the presence or absence of LPS (1 *μ*g/ml) for 6 h. Total RNA was isolated from the RAW264.7 cells using TRIzol reagent according to the manufacturer's instructions, and 1 *μ*g of total RNA was used for cDNA synthesis with a cDNA synthesis kit (Thermo Fisher Scientific, Waltham, MA, USA). Total RNA was stored at −70°C until use. Semiquantitative RT-PCR reactions were conducted as previously reported [16]. All the primers used in this study are shown in Table 2.

### 2.7. Plasmid Transfection and Luciferase Reporter Gene Assay

RAW264.7 and HEK293 cells (1x10^6^ cells/ml) were transfected with 1.1 *μ*g of plasmid encoding *β*-galactosidase and NF-*κ*B-luc in the presence or absence of an inducing molecule (Flag-MyD88, CFP-TRIF, Myc-Syk, or HA-Src) using the PEI method for 24 hours. The cells were then treated with Ac-ME at the indicated concentration for 24 hours. The cell aliquots were treated with either *β*-galactosidase or luciferase buffer to measure the absorbance and luminescence, respectively. Luciferase assays were conducted using the Luciferase Assay System (Promega, Madison, WI, USA) as previously reported [11, 17].

### 2.8. Preparation of Total Lysates and Immunoblotting

RAW264.7 or HEK293 cells were plated with a density of 2 × 10^6^ cells/ml, preincubated for 18 h, and then treated with Ac-ME (100 or 200 *μ*g/ml) in the presence or absence of LPS (1 *μ*g/ml) for indicated times. Ac-ME-treated RAW264.7 and HEK293 cells were harvested, washed in cold PBS, and lysed in lysis buffer [20 mM Tris-HCl (pH 7.4), 20 mM NaF, 2 mM EDTA, 2 mM EGTA, 50 mM *β*-glycerol phosphate, 1 mM DTT, 2 *μ*g/ml aprotinin, 2 *μ*g/ml leupeptin, 1 *μ*g/ml pepstatin, 50 mM PSMF, 1 mM benzamide, 2% triton X-100, 10% glycerol, 0.1 mM sodium vanadate, and 1.6 mM pervanadate] for 30 minutes. The lysates were then cleared by centrifugation at 12000xg for 10 minutes at 4°C, and the supernatants were stored at −20°C. The whole cells were analyzed using immunoblotting as previously reported [18]. Densitometric scanning values of each protein from blots were obtained using the DNR Bio-imaging system (a Gelquant software Ver. 2.7, Neve Yamin, Israel). Calculation of relative intensity was carried out with the following equation. Relative intensity = densitometric scanning value of phosphoproteins or total proteins/densitometric scanning value of corresponding total protein or loading control (*β*-actin).

### 2.9. Statistical Analysis

Data are expressed as mean ± standard deviation (SD) of at least three independent experiments performed in triplicate or of one representative experiment out of three different experiments with similar results. The results were interpreted using either ANOVA/Scheffe's* post hoc* test or the Kruskal-Wallis/Mann-Whitney test for statistical comparisons. The data was statistically significantly different if p<0.05. All statistical tests were carried out using the computer program SPSS (SPSS Inc., Chicago, IL, USA).

## 3. Results

### 3.1. Effect of Ac-ME on Inflammatory Responses

**Figure 1:**
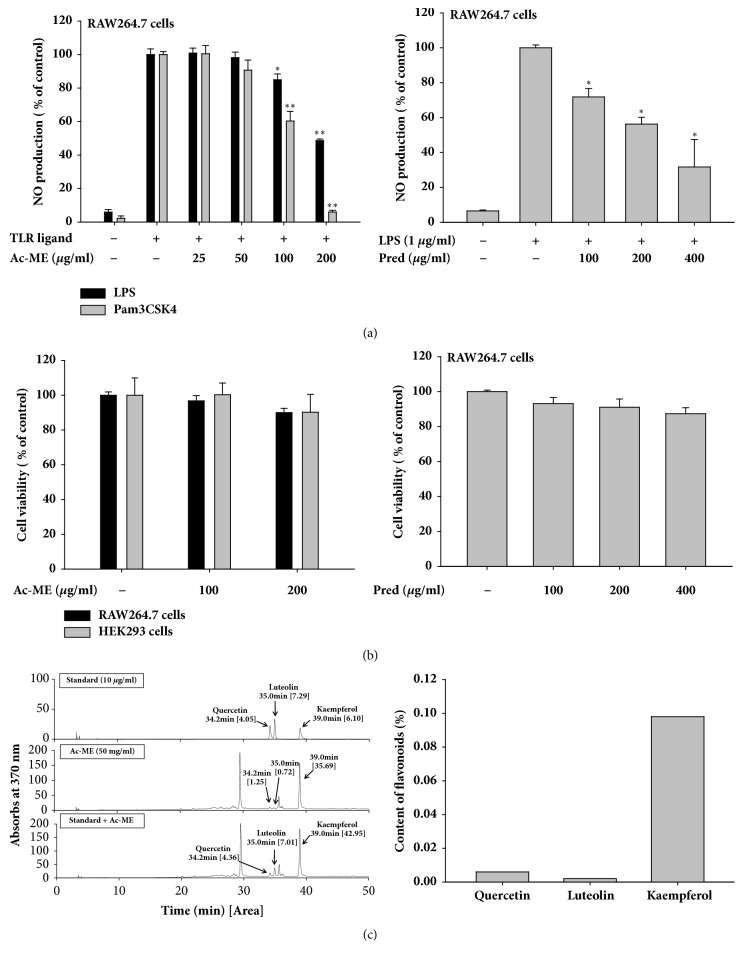
Effect of Ac-ME on production of inflammatory mediators and HPLC profiling of Ac-ME. (a) Levels of NO production induced by Pam3CSK4 (a TLR2 ligand) and LPS (a TLR 4 ligand) were determined by the Griess Assay from supernatant of RAW264.7 cell treated with Ac-ME (left panel) or prednisolone (Pred) (right panel) for 24 h. (b) Cell viability of RAW264.7 and HEK293 cells treated with Ac-ME (left panel) or Pred (right panel) was determined by MTT assay. (c) The phytochemical profile of Ac-ME was analyzed by HPLC with the conditions described in Table 1 (left panel). The content of flavonoids was shown in the graph by calculating from the standard curves of the compounds (right panel). *∗P* < 0.05 and *∗∗P *< 0.01 compared to the control.

To investigate whether Ac-ME has an effect on modulating the inflammatory response, the NO (nitric oxide) level of the LPS-treated RAW264.7 cells (TLR 4-ligand) was determined. As shown in Figure 1(a) left panel, Ac-ME dose-dependently decreased the level of NO production to 48.62 ± 0.99% at 200 *μ*g/ml. Similarly, it also diminished the production of NO from Pam3CSK4-treated RAW264.7 cells (TLR 2-Ligand) to 5.97 ± 1.07% at 200 *μ*g/ml. Standard anti-inflammatory compounds such as prednisolone demonstrated similar activity in inhibiting NO level. In Figure 1(a) right panel, prednisolone dose-dependently suppressed the NO level to 31.68 ± 15.74% at 400 *μ*g/ml. From Figures 1(b) left panel and 1(b) right panel, no cell toxicity was observed in the RAW264.7 and HEK293 cells, indicating that the NO inhibitory effects were not due to the toxic activity of the compounds.

Ac-ME were analyzed using HPLC analysis to identify its anti-inflammatory constituents. For this, we selected quercetin, luteolin, and kaempferol, because not only these compounds were found to suppress macrophage-mediated inflammatory responses [19–21], but also these compounds were identified in Genus* Abutilon* [22]. As shown in Figure 1(c) left panel, the components of Ac-ME were analyzed with the standard anti-inflammatory compounds quercetin, luteolin, and kaempferol. In Figure 1(c) right panel, the HPLC data showed that Ac-ME contains 0.098% of kaempferol, 0.006& of quercetin, and 0.002% of luteolin.

### 3.2. Ac-ME Suppresses Inflammatory Responses at the Transcriptional Level

**Figure 2:**
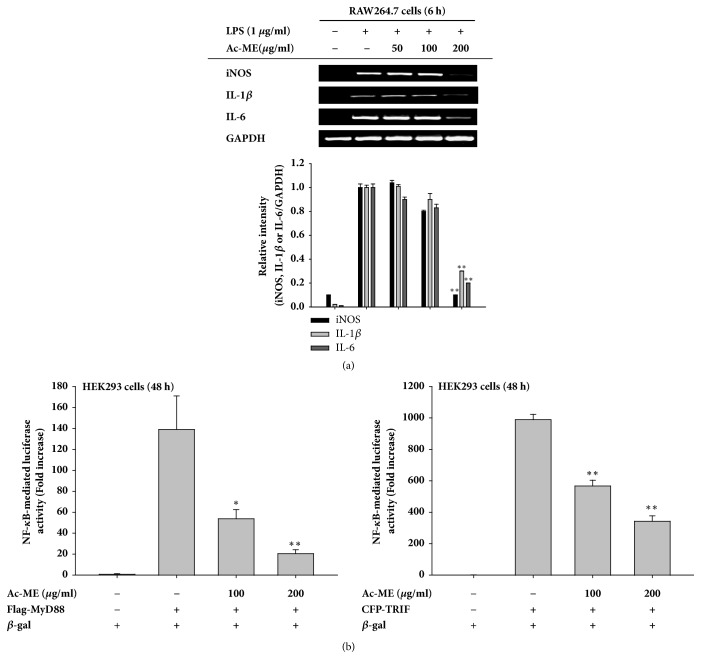
Effect of Ac-ME on the transcriptional regulation of inflammatory genes. (a) Using semiquantitative RT-PCR, the mRNA levels of iNOS, IL-1*β*, and IL-6 were determined (upper panel). Relative intensity (lower panel) was values of ratio calculated using densitometric scanning values of inflammatory genes (iNOS, IL-1*β* or IL-6) and densitometric scanning values of GAPDH by the DNR Bio-imaging system (a Gelquant software Ver. 2.7). (b) HEK293 cells were cotransfected with MyD88 (left panel) and TRIF (right panel). The luciferase activity was measured using a luminometer. *∗P* < 0.05 and *∗∗P *< 0.01 compared to the control.

mRNA levels were examined to determine whether Ac-ME can modulate inflammation at the transcriptional or translational level. As seen in Figure 2(a), LPS treatment increased the levels of iNOS, COX-2, TNF-*α*, IL-1*β*, and IL-6. Ac-ME suppressed the upregulation level of iNOS, IL-6, and IL-1*β* at 200 *μ*g/ml, which indicates an effect on transcription. The regulatory role of Ac-ME on the activation of inflammatory transcription factors was determined by the luciferase assay. HEK293 cells were cotransfected with myeloid differentiation primary response 88 (MyD88) and TIR-domain-containing adapter-inducing interferon-*β* (TRIF) together with NF-*κ*B luciferase gene. Treatment of Ac-ME to the transfected cells inhibited the NF-*κ*B mediated luciferase activity in a dose-dependent manner up to 20.43% and 66.89% at 200 *μ*g/ml, respectively, shown in Figure 2(b) left panel and right panel.

### 3.3. Ac-ME Inhibits the NF-*κ*B Activation Pathway

**Figure 3:**
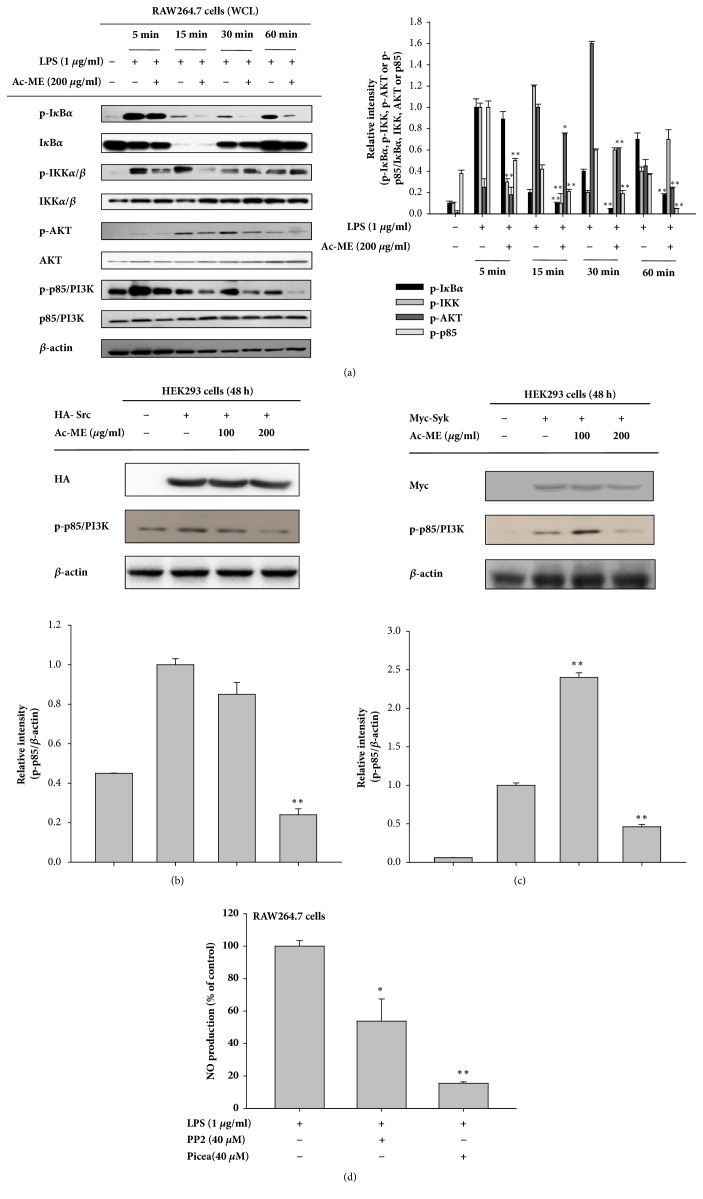
Effect of Ac-ME on the upstream signaling of the NF-*κ*B pathway. Immunoblotting was performed to determine the target of Ac-ME in modulating the inflammatory response. ((a) left panel) Phosphoprotein and total protein levels of I*κ*B*α*, IKK*α*/*β*, AKT, and p85/PI3K were determined by immunoblotting analysis with specific antibodies. ((a) right panel) Relative intensity was values of ratio calculated using densitometric scanning values of p-I*κ*B*α*, p-IKK*α*/*β*, p-AKT, or p-p85 and densitometric scanning values of their total proteins (I*κ*B*α*, IKK*α*/*β*, AKT, or p85) by the DNR Bio-imaging system (a Gelquant software Ver. 2.7). (b, c) HEK293 cells were transfected with Myc-Syk or HA-Src and, at 36 h, Ac-ME (100 – 200 *μ*g/ml) was treated to the transfected cells for further 12 h. The levels of phospho-p85, *β*-actin, Myc, or HA were identified by immunoblotting analysis. Relative intensity was values of ratio calculated using densitometric scanning value of p-p85 and densitometric scanning value of *β*-actin by the DNR Bio-imaging system (a Gelquant software Ver. 2.7). (d) The NO inhibitory levels of Syk and Src inhibitors (PP2 and Piceatannol) were measured with LPS-treated RAW264.7 cells. *∗P* < 0.05 and *∗∗P *< 0.01 compared to the control.

To identify the exact target of Ac-ME, we conducted immunoblotting analysis to determine which proteins are stopped in the cascade of the inflammatory response. As we expected and as shown in Figure 3(a) (left and right panels), Ac-ME (200 *μ*g/ml) clearly suppressed the level of p-I*κ*B*α* upregulated by LPS at 5, 15, 30, and 60 min. Through the analysis of upstream signaling, it was found that the phosphorylation of p85/PI3K was significantly blocked by this extract at 5, 15, 30, and 60 minutes. Overexpression of its upstream kinases (Syk and Src) was performed to observe the involvement of these two proteins in Ac-ME-mediated anti-inflammatory action. Ac-ME did not inhibit either Src or Syk, but as shown in Figures 3(b) and 3(c) left panel and right panel, p-PI3K was blocked by Ac-ME both in Src and Syk overexpression. Finally, NO production was also strongly diminished by specific inhibitors (PP2 and Piceatannol) of Src and Syk, indicating that these enzymes play critical roles in inflammatory responses in LPS-treated macrophages (Figure 3(d)).

## 4. Discussion

Natural remedies are becoming popular in the pharmaceutical field and market due to the desire for fewer synthetic ingredients and side effects. Therefore, the development of plant-derived drugs is preferable in the research field nowadays.* Abutilon crispum *L. Medik is one of the plants whose function has yet to be widely known. However, some papers previously reported that* Abutilon crispum* has an anti-inflammatory effect [10], but the molecular mechanism behind it has not been revealed. Therefore, in this study, we investigated the anti-inflammatory effect of Ac-ME regarding the NF-*κ*B pathway.

First, we performed the NO assay to determine whether Ac-ME can inhibit the production of inflammatory mediators. NO or nitric oxide is one of the inflammatory mediators or signaling molecules that can induce inflammation when overproduced [23]. To induce NO production, LPS and Pam3CSK4 were used as inducers in the NO assay. LPS and Pam3CSK4 are molecule-derived pathogens or PAMPs that can be recognized by TLRs and activate the signaling cascade to produce inflammatory cytokines and a resulting inflammatory response [24]. LPS or lipopolysaccharide is derived from the outer surface membrane component in gram-negative bacteria and induces a strong innate immunity in humans [25], while Pam3CSK4 is a synthetic tripalmitoylated lipopeptide that can mimic the acetylated amino terminus of bacterial lipoproteins and can be recognized by TLR 2 [26]. From the results shown in Figure 1(a), Pam3CSK4 and LPS can dose-dependently downregulate the production of NO treated by Ac-ME. To confirm the pattern of Ac-ME inhibiting NO production, we also assessed an anti-inflammatory drug, prednisolone. Prednisolone is a corticosteroid drug that suppresses inflammatory genes by reversing histone acetylation-activated inflammatory genes through the binding of liganded glucocorticoid receptors (GR) to coactivators and recruitment of histone deacetylase-2 (HDAC2) to the activated transcription complex [27].  Figure 1(b) shows that the prednisolone group dose-dependently inhibited the level of NO production. Prednisolone exhibits the same pattern in downregulating the level of NO production as Ac-ME. Therefore, from the NO data, we conclude that Ac-ME has anti-inflammatory activity. We also must confirm that this result is not due to the toxicity of the compound to the cell. As a result, we assessed cell viability by performing the MTT assay with RAW264.7 and HEK293 cells. In the assay, the compound (Figure 1(b) (left panel)) did not exhibit any toxicity in either cell lines or the group treated with prednisolone (Figure 1(b) (right panel)). We also attempted to identify the components in Ac-ME which contribute to its anti-inflammatory activity. HPLC analysis was done with standard compounds of anti-inflammatory flavonoids including luteolin, quercetin, and kaempferol [28]. In Figure 1(c) right panel, it is shown that kaempferol has the highest content among others. So far, few information of phytochemical feature of this plant has been revealed. Thus, it was reported that *β*-sitosterol, lupeol, and *β*-amyrin are contained in the leaves of this plant [29]. In addition, genus* Abutilon* has been reported to contain flavonoids, phenolic acids, sterols, triterpenes, coumarins, alkaloids, lactones, megastigmanes, and iridoids [22]. With kaempferol that we have identified in this study, a lot of compounds including caffeic acid, rutin, myricetin, catechin, benzoic acid, *β*-amyrin, lupeol, and *β*-sitosterol have been known as anti-inflammatory components [30–32]. Therefore, some of these compounds included in Ac-ME could contribute to the anti-inflammatory activity of Ac-ME. By activity-guided fractionation, we will further define additional anti-inflammatory constituents from Ac-ME in the following project.

When inflammation occurs, NO is produced by inducible nitric oxide synthase (iNOS) in the macrophages [33]. iNOS is responsible for the roles of NO in the inflammatory response [34]. Other genes also play an important role in interceding inflammation. Therefore, we analyzed the mRNA expression of inflammatory genes by performing a reverse transcriptase polymerase chain reaction (RT-PCR). iNOS, IL-1*β*, and IL-6 were investigated with RT-PCR, and the expression of these genes were inhibited by Ac-ME at 200 *μ*g/ml, as shown in Figure 2(a) left panel and right panel.

To confirm whether Ac-ME is capable of modulating the activation of transcription factors, we performed the luciferase reporter gene assay. Cells were cotransfected with TRIF (TIR-domain containing adapter-inducing interferon-b) and MyD88 (myeloid differentiation primary response), an adaptor protein that can induce NF-*κ*B-mediated luciferase activity. When the TLR binds with its ligand, the receptor will recruit the adaptor protein so that the cascade will occur [35]. Figures 2(b) left panel and 2(b) right panel show that Ac-ME can alter NF-*κ*B-mediated luciferase activity. Therefore, we continued to search for the target of Ac-ME upstream.

**Figure 4:**
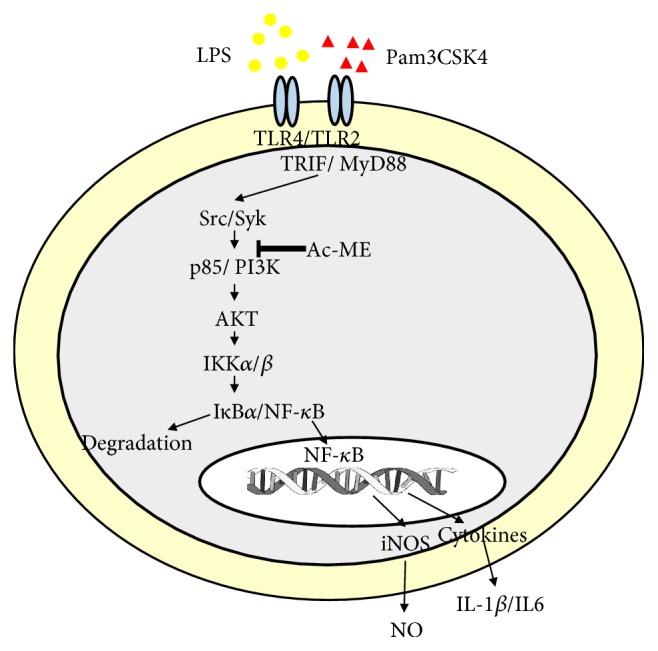
Putative inhibitory pathway of Ac-ME in mediating the inflammatory response. It is assumed that Ac-ME targets the activation of both Src and Syk linked to the activation of intracellular signaling pathway for the nuclear translocation of NF-*κ*B. Suppression of this pathway leads to the downregulation of iNOS-mediated NO production and the expression of proinflammatory cytokines such as IL-1*β* and IL-6.

We performed immunoblotting assay with western blot analysis. Figure 3(a) shows that the phospho-p85, a regulatory subunit of PI3K, was inhibited by Ac-ME after 5 min of cells exposure to LPS. Therefore, we measured the overexpression strategies by overexpressing the tyrosine kinases, Syk and Src, in HEK293 cells. In Figures 3(b) and 3(c) left panel and right panel, both p-p85 proteins were inhibited by Ac-ME with the overexpression of Syk and Src. Figure 3(d) shows the inhibitory level of NO production when treated with PP2 and Piceatannol, inhibitors of Syk and Src, respectively. There was a significant difference between the control group and the inhibitor-treated group. These results strongly indicate that the anti-inflammatory effects of Ac-ME are mediated through the suppression of NF-*κ*B as a result of inhibition of PI3K (Figure 4). Further studies are required to understand how Ac-ME can inhibit PI3K in the inflammatory response and to discover other possible targets of Ac-ME.

## 5. Conclusion

The study showed that the Ac-ME has an anti-inflammatory effect in the NF-*κ*B pathway by inhibiting the phosphorylation of p85/PI3K.

## Figures and Tables

**Table 1:** HPLC condition for analyzing Ac-ME constituents with quercetin, luteolin, and kaempferol as standard compounds.

Instrument	KNAUER Corp. HPLC system		
Column	Phenomenex, Gemini 5 *μ*m C18 100A, 250 × 4.60 mm 5 mm		
Column temperature	35°C		
Detector	UV/VIS detector 370 nm		
Analysis software	ClarityChrom Software		
Injection volume	20 *μ*l		
Standard	Diluted with DMSO		
Sample treatment	50 mg/ml, diluted with DMSO		
Mobile phase			
Solvent A	0.1% TFA in H_2_O		
Solvent B	0.08% TFA in 95% MeCN + 5% H_2_O		
Flow rate	1.0 ml/min		
Time table	Time (min)	Solvent A (%)	Solvent B(%)
	0	100	0
	50	50	50
	60	0	100

**Table 2:** Primer sequences used in PCR analysis.

Gene Target		Sequence (5′-3′)
iNOS	F	GTG AAG AAA ACC CCT TGT GCT G
	R	AGT TCC GAG CGT CAA AGA CC
IL-1*β*	F	CAG GAT GAG GAC ATG AGC ACC
	R	CTC TGC AGA CTC AAA CTC CAC
IL-6	F	GCC TTC TTG GGA CTG ATG CT
	R	TGG AAA TTG GGG TAG GAA GGA C
GADPH	F	ACC ACA GTC CAT GCC ATC AC
	R	CCA CCA CCC TGT TGC TGT AG

## Data Availability

The data used to support the findings of this study are available from the corresponding author upon request.

## Abbreviations

HEK: Human embryonic kidney
COX: Cyclooxygenase
TNF-*α*: Tumor necrosis-alpha
IL: Interleukin
GAPDH: Glyceraldehyde 3-phosphate dehydrogenase
I*κ*B*α*: Inhibitor of kappa B alpha
IKK: I*κ*B kinase
NF-*κ*B: Nuclear factor-kappa B
AP-1: Activator protein-1
IRF-3: Interferon regulatory factor-3
Picea: Piceatannol
Pred: Prednisolone
FBS: Fetal bovine serum.
